# Nanostructured Composites Based on Liquid-Crystalline Elastomers

**DOI:** 10.3390/polym10070773

**Published:** 2018-07-14

**Authors:** Vanessa Cresta, Giuseppe Romano, Alexej Kolpak, Boštjan Zalar, Valentina Domenici

**Affiliations:** 1 Dipartimento di Chimica e Chimica Industriale, Università di Pisa, via Moruzzi 13, 56124 Pisa, Italy; vane.cresta@hotmail.it; 2 Department of Mechanical Engineering, Massachusetts Institute of Technology, 77 Massachusetts Avenue, Cambridge, MA 02139-4307, USA; romanog@mit.edu (G.R.); kolpak@mit.edu (A.K.); 3 Department of Condensed Matter Physics, Jozef Stefan Institute, Jamova Cesta 39, SI 1000 Ljubljana, Slovenia; bostjan.zalar@ijs.si

**Keywords:** liquid-crystal polymers, bilayers, composites, liquid single-crystal elastomers, actuators, artificial muscles, orientational order, NMR, nanoparticles, nanomaterials, photo-actuation, electro-actuation, thermal actuation

## Abstract

Liquid-crystalline elastomers (LCEs) are the object of many research investigations due to their reversible and controllable shape deformations, and their high potential for use in the field of soft robots and artificial muscles. This review focuses on recent studies about polymer composites based on LCEs and nanomaterials having different chemistry and morphology, with the aim of instilling new physical properties into LCEs. The synthesis, physico-chemical characterization, actuation properties, and applications of LCE-based composites reported in the literature are reviewed. Several cases are discussed: (1) the addition of various carbon nanomaterials to LCEs, from carbon black to carbon nanotubes, to the recent attempts to include graphene layers to enhance the thermo-mechanic properties of LCEs; (2) the use of various types of nanoparticles, such as ferroelectric ceramics, gold nanoparticles, conductive molybdenum-oxide nanowires, and magnetic iron-oxide nanoparticles, to induce electro-actuation, magnetic-actuation, or photo-actuation into the LCE-based composites; (3) the deposition on LCE surfaces of thin layers of conductive materials (i.e., conductive polymers and gold nanolayers) to produce bending actuation by applying on/off voltage cycles or surface-wrinkling phenomena in view of tunable optical applications. Some future perspectives of this field of soft materials conclude the review.

## 1. Introduction

**Scheme 1:**
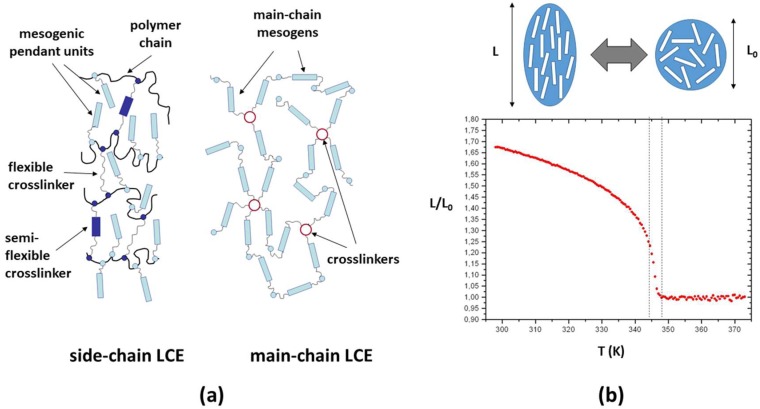
(**a**) Sketch of the main constituents of a side-chain liquid-crystalline elastomer (LCE; on the left) and of a main-chain LCE (on the right). (**b**) Top: scheme of the volume deformation of a typical LCE system at the phase transition from a spherical shape (disordered phase) to an oblate uniaxial shape (ordered phase). Bottom: the uniaxial elongation, reported as the ratio L/L_0_ versus temperature (T) of a typical nematic LCE.

Liquid-crystal polymers (LCPs) are a class of materials that combine the mesophasic features of low-molar-mass liquid crystals with the versatile physical properties of polymers [1]. A liquid-crystal polymer not only possesses the individual properties of each of its constituents, but also exhibits intrinsic new features. LCPs may exhibit either nematic and/or smectic phases, depending on the chemical structure of the mesogenic molecules, the flexible spacers, and the polymer main chains [2]. Moreover, the mesomorphic behavior of LCPs can be influenced by external physical parameters, such as temperature and pressure [3]. Liquid-crystalline elastomers (LCEs) are a particular type of LCPs, where the polymer chains are cross-linked together via flexible or semi-flexible cross-linkers [4]. As in the case of LCPs, side-chain and main-chain systems can be distinguished: side-chain LCEs contain liquid-crystalline molecules as pendant units, while main-chain LCEs constituted mesogenic moieties as a part of the main-chain polymer backbone (Scheme 1a). As reported in several books and reviews [5,6,7,8,9,10,11,12,13], the interplay between the elasticity of the polymer network and the spontaneous orientational ordering of the liquid-crystalline units results in a reversible thermo-mechanical response, as first predicted by de Gennes in 1975 [14] (Scheme 1b). Several synthetic strategies were developed in order to obtain macroscopically aligned LCE samples [6,15,16,17,18,19,20]. The preparation of macroscopically aligned LCEs, associated with a relatively homogeneous orientational order (*S*) of the local nematic (or smectic) domains, is indeed a fundamental feature for their peculiar physical properties, such as the macroscopic, controllable, and reversible shape changes [21]. LCEs are included in a type of materials called “shape-memory” materials, due to their ability to memorize their macroscopic shape under specific conditions. 

In common macroscopically aligned (monodomain) LCEs, mechanical deformation is induced by increasing (or decreasing) the temperature across the disordered–ordered phase transition and vice versa (Scheme 1b) [15]. However, the perspectives of applications of these materials could be widely expanded if the reversible shape variation was induced by stimuli other than temperature, such as, for instance, external electric or magnetic fields, as in common electro-active polymers [22,23,24]. Another possibility, explored by several research groups, is the preparation of photosensitive LCEs, in order to modulate the shape deformation and morphing geometries by means of UV-vis or infrared (IR) light stimuli [25,26,27,28,29]. These are the two main directions of development in the field of LCE-based materials. 

In this review, the focus is on the actual state of the art concerning the preparation, the characterization, and the actuation properties of composite materials based on LCEs. In fact, this field is the object of great expansion, and an increasing number of works was published in the past five years. In particular, the main studies about LCEs prepared, or reprocessed, with micro- and nanostructured materials [30] are reviewed, as well as composite bilayers made of LCE films covered by conductive materials in the form of thin films. Some future perspectives of functional nanostructured LCE composites are discussed in view of their potential applications, such as artificial muscles, smart surfaces, micro-electromechanical systems (MEMS), and nano-electromechanical systems (NEMS) [31,32,33,34]. 

## 2. LCE-Based Composites with Carbon Micro- and Nanostructured Materials 

The induction of electro-mechanical actuation in LCE matrices by applying external stimuli, other than thermal actuation, was the purpose of many researches in the past 15 years. The first attempts concerned the preparation of composites made from carbon materials, and macroscopically aligned liquid-crystalline elastomers were obtained according to the so-called “two-step” cross-linking procedure, first described by Kupfer et al. [35]. Various types of carbon materials are used to prepare these LCE-based composites, such as carbon micro- and nanoparticles [36,37,38,39], carbon nanotubes (CNT) [40,41,42,43,44], fullerene (C60) derivatives [45], and graphene and its derivatives [45,46,47,48]. In the following sections, details about the preparation, physico-chemical characterization, and actuation properties are reported and discussed.

### 2.1. Sample Preparation and Characterization of Carbon-Based LCE Composites 

Carbon micro- and nanomaterials are considered good candidates as polymer dopants due to their conductivity, adhesion, wide range of morphologies and sizes, and for their relatively low cost [49]. Most LCE-based composites are made by macroscopically aligned monodomain LCE films, prepared following the “two-step” cross-linking procedure [35]. These films are also named liquid single-crystal elastomers (LSCEs) to distinguish them from all other kinds of LCEs. Their preparation includes a first step involving a cross-linking reaction where a pre-polymerization mixture of the polymer main chains, monomers, and cross-linkers in a solvent (e.g., toluene) are put inside a rotor with a known and controlled temperature and rate until a gel-like film is obtained. The second step of the cross-linking reaction occurs by handing the gel-like film under progressive weights in order to align the monomers along the longitudinal direction of the films [35]. This procedure gives rise to uniaxially aligned LCE films with very reproducible thermo-mechanical and ordering properties [16]. Most known LSCE systems are formed starting from polysiloxane-based chains, and, depending on the chemistry, they can form ordered mesophases (typically the nematic phase), stable at room temperature. In order to induce a response to electric, magnetic, and/or light stimuli in standard LSCEs, which are highly insulating, various procedures were adopted.

One method was proposed by Chambers et al. [36,37,38,39] with carbon-black spherical nanoparticles, with an average diameter of 30 nm, and carbon nanohorns of about 30–50 nm in length and 4 nm in diameter. Standard monodomain LCE films, namely LSCEs, were swollen in different solvents (methanol, cyclohexane, and toluene) with a concentration of nanoparticles ranging from 0.05 g/L to 5 g/L [36]. According to this swelling procedure [36,37], the carbon nanomaterials, which are dispersed in the solvents, are able to partially penetrate the LSCE matrix only in the gel-like swollen state. The optimal penetration depth and nanocarbon layer thickness were reached with a mixture of cyclohexane/toluene, and it was found to be a few micrometers. The process of absorption/desorption of the nanoparticles to the swollen LSCE films was studied by applying the Langmuir theory [50], and the conductivity of the obtained LSCE-based composites in the swollen state was evaluated through their effective resistivity (ρ, Ω/cm). 

**Figure 1:**
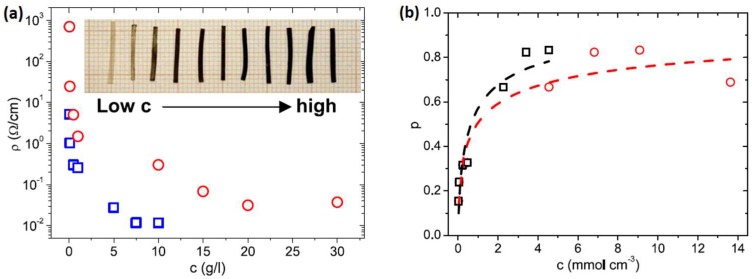
Liquid single-crystal elastomer (LSCE) reprocessed with carbon nanoparticles. (**a**) Effective resistivity (ρ) of the macroscopic LSCE as a function of concentration (c) for carbon black (blue squares) and carbon nanohorn (red circles) particles of a reprocessed side-chain siloxane-based LSCE film. Top: picture of carbon-black-reprocessed LSCE samples. (**b**) Particle fraction (p) versus concentration (c) for carbon black (black squares) and carbon nanohorns (red circles). The best-fit lines of the Langmuir equation are also shown [50]. Adapted with permission from the authors of [37]. © IOP Publishing. Reproduced with permission. All rights reserved.

As observed in Figure 1a, a minimum in resistivity, associated with a saturation of nanocarbon absorption, was observed around 7.5 g/L and 20 g/L [37] in LSCEs reprocessed with carbon black and carbon nanohorns, respectively. The swelling procedure described by Chambers et al. [36,37] produces LSCE films with integrated external layers of various thicknesses with a volume fraction of carbon nanoparticles (*p*) which is dependent on the initial nanoparticle concentration (c; Figure 1b). The mechanism of swelling/deswelling of both LCEs and LCE composites was the object of several experimental and theoretical investigations [51,52,53,54,55]. Most of them refer to pristine liquid-crystalline elastomers swelled in appropriate low-molecular-mass LCs (LMMLCs) to study different geometrical distortions of the nematic director, electrical deformations, and electro-optical and mechanical properties [51]. Torsional actuation was studied in hierarchically patterned LCE materials under the effect of chemical vapor in view of applications as chemo-responsive systems [52]. LCEs swollen with nematic solvents featuring different swelling ratios still retained their main elastic properties, such as the soft and semisoft elasticity typical of LCEs [53]. Recent theoretical investigations [54,55] confirmed these findings showing potential applications of swollen LCEs as sensors, actuators, and micro-swimmers. Few studies, however, shed light on swollen LCE composites [36,37,38,39]. 

An alternative approach to producing LCE-based composites was reported by Marshall et al. [40] using carbon nanotubes (CNTs), which are able to absorb light in a wide range of wavelengths, and which can convert light into heat. The procedure described by the authors of [40] consists of a functionalization of the CNTs with pirene-ending polymer chains acting as a dispersing agent to avoid the aggregation of CNTs. The functionalized CNTs are then dispersed in toluene at a known concentration, and the pre-polymerization constituents (polysiloxane chains, mesogens, and cross-linkers) are added. The obtained dispersion with all starting reactants is then introduced in the rotor following the “two-step” cross-linking procedure [35]. Unlike Chambers’s swelling method [36,37,38,39], in this approach [40], the carbon nanomaterials are added before the preparation of the LSCE films. As a main result, the distribution of the functionalized CNTs within the LSCE matrix is more homogeneous in the volume with respect to the previous method. As in many other cases, the concentration of the nanomaterials is rather crucial in determining the physical properties of the LCE-based composites, as discussed in the later sections. 

**Figure 2:**
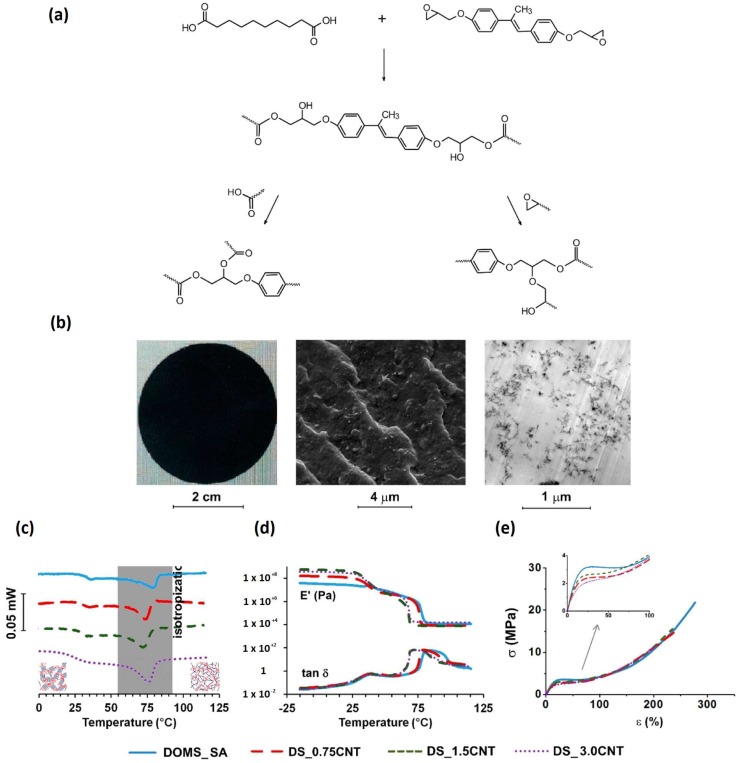
(**a**) Scheme of the synthetic procedure to prepare smectic epoxy-based elastomer film samples (named DOMS_SA). (**b**) Optical image (left), SEM micrograph (center), and bright-field TEM image (right) of a smectic film doped with multi-walled carbon nanotubes (sample DS_1.5CNT). (**c**) Differential scanning calorimetry (DSC) curves of the investigated samples. (**d**) Storage modulus (E’) and tan δ of the investigated samples. (**e**) Stress–strain curves (σ versus ε%) of the investigated samples. Adapted with permission from the authors of [44]. Copyright (2018) American Chemical Society.

A completely different synthetic procedure was reported by Lama et al. [44] to produce epoxy-based LCE composites with multi-walled carbon nanotubes (MWCNTs). The synthesis of the epoxy polymer (labeled “DOMS_SA”, Figure 2a) involves the mixing of the epoxy precursor with sebacic acid in the presence of a catalyst, obtaining a viscous mixture at 160 °C. The mixture is then poured between two Teflon-coated glasses, and is cured in the oven to finalize the polymerization. The synthesis of epoxy-based LCE-MWCNT composites implies the functionalization of the MWCNTs with the epoxy pre-polymer. The curing reaction is then performed by adding sebacic acid to the dispersion of epoxy-functionalized MWCNTs in the oven, as described for the pure epoxy polymer [44]. Several samples in the form of thick plates (10 mm × 10 mm × 0.25 mm) were obtained with varying nanotube content, indicated as DS_xCNT, with x = 0.75 wt %, 1.5 wt % and 3.0 wt %. An important step in the preparation of homogeneous LCE composites is represented by the functionalization of the MWCNTs and the formation of the epoxy precursor–MWCNT adducts, as revealed by the optical, SEM and TEM images (Figure 2b). The mesophase behavior of the LCE and LCE-based composites indicates the occurrence of a smectic phase (see differential scanning calorimetry (DSC) in Figure 2c). However, a concentration, x, of MWCNTs higher than 1.5 wt% destabilizes the mesomorphic behavior, as observed in other LCE composites [37,40,41,42,43,44]. As shown in Figure 1d and Figure 1e, both dynamic mechanical analysis and stress–strain measurements confirm the physical properties typical of liquid-crystalline elastomers. The storage modulus (*E’*) and dissipation factor (*tan δ*) indicate an increasing stiffness of the LCE composites when the concentration of MWCNT is increased (Figure 1d). Stress–strain curves (σ versus ε%) refer to the uniaxial mechanical stress applied to the rubber state of the samples, which is able to align the network. The typical soft-elasticity response of LCEs is also observed in the composites (Figure 1e). As reported and discussed in Reference [44], the presence of MWCNTs has interesting effects on the thermo-mechanical behavior. In particular, at x = 0.75 wt%, the LCE-based composites show a dramatic enhancement in thermal actuation and a very high orientational order, probably due to alignment of the nanotubes along the smectic director. 

**Figure 3:**
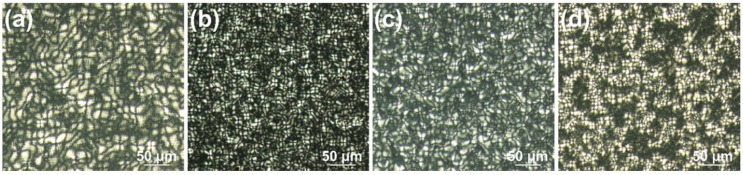
Liquid-crystalline polyesters reprocessed with various carbon nanomaterials. Polarized optical microscopy (POM) photographs captured at 30 °C: (**a**) PBDPS, (**b**) PC60 (0.5%), (**c**) PCNT (0.5%), and (**d**) PG (0.5%). Labels: PBDPS, phenyl succinate-based main chain LC polymer; PC60, composite sample made of PBDPS including fullerene C60; PCNT, composite sample made of PBDPS including carbon nanotubes; PG, composite sample made of PBDPS including graphene. Reproduced with permission from the authors of [45]. Copyright © 2018 Elsevier.

**Figure 4:**
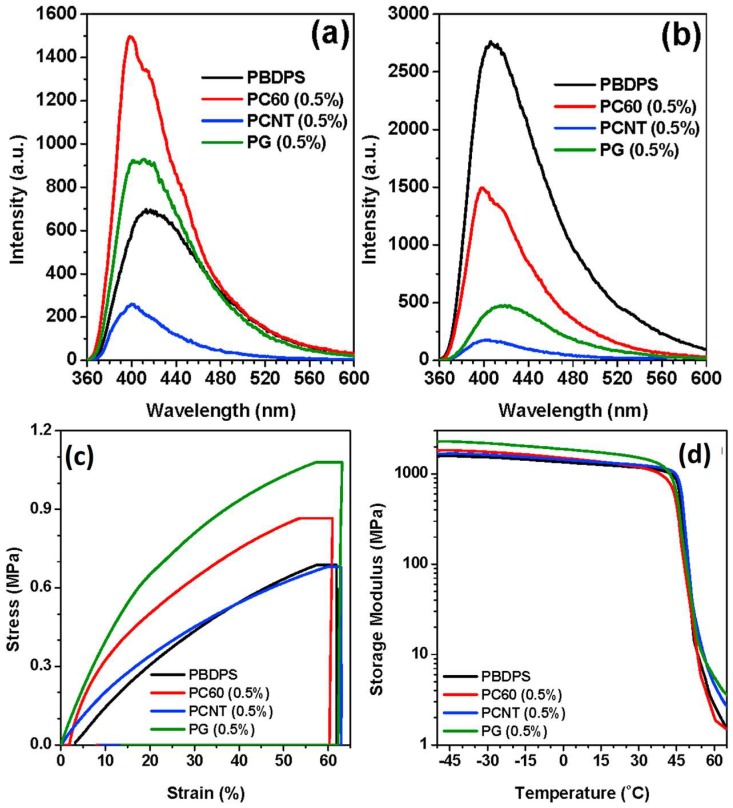
Liquid-crystalline polyesters reprocessed with various carbon nanomaterials. Fluorescence spectra of PBDPS and PBDPS/carbon nanofiller composites at concentrations of 1 mg/mL (**a**) and 5 mg/mL (**b**). Dynamic mechanical results of the testing samples: (**c**) recovery stress–strain curves at 40 °C; (**d**) storage modulus as a function of temperature. Labels: PBDPS, phenyl succinate-based main chain LC polymer; PC60, composite sample made of PBDPS including fullerene C60; PCNT, composite sample made of PBDPS including carbon nanotubes; PG, composite sample made of PBDPS including graphene. Adapted with permission from the authors of [45]. Copyright © 2018 Elsevier.

In addition to carbon nanoparticles and carbon nanotubes, recent studies reported the preparation of liquid-crystalline elastomers with graphene [45,46,47] and fullerene derivatives [45]. An original synthetic approach was proposed by Meng et al. [45] to produce shape-memory elastomers without chemical cross-linkers, which are substituted by physically cross-linker points. These materials are main-chain liquid-crystalline polymers (labeled PBDPS, Figure 3) obtained using biphenyl-phenyl succinate derivatives, where the adjacent phenyl rings are stacked together forming strong π–π interactions [45]. PBDPS exhibits physical properties typical of chemically cross-linked LC polymers, such as a large maximum strain (>220%) and shape-recovery ratio (>99%). The addition of low concentrations of carbon nanomaterials (~0.5 wt%) was investigated by means of several physico-chemical techniques, such as TEM, polarized optical microscopy (POM; Figure 3), X-ray, fluorescence spectroscopy (Figure 4a,b), and dynamic mechanical analysis (Figure 4c,d). 

The liquid-crystalline textures of the pristine elastomer matrix, named PBDPS, and the corresponding LCE composites containing 0.5 wt% of the carbon-based nanomaterials can be characterized using POM, as reported in Figure 3. The smectic A phase of the LCP gives rise to typical Schliren textures with fourfold singularities. The presence of the carbon nanomaterials sensibly does not change the textures, thus indicating that the birefringence properties are retained [45].

The fluorescence emission of PBDPS doped with small concentrations of carbon nanofillers, in particular C60 and graphene (see Figure 4a), is enhanced, thus confirming that the presence of nanomaterials acts as an additional physical cross-link of the polymer network. At higher concentrations, the quenching phenomena are more pronounced due to the energy transfer activated by the strong interactions between the nanocarbon materials and the π–π groups. This effect is also evident from dynamic mechanical analysis, showing that the addition of CNTs, C60, or graphene (at low wt%) increases the recovery stress (Figure 4c) and the storage modulus (Figure 4d), especially in the case of graphene. When the concentration of graphene is higher than 0.5 wt%, however, the effect is opposite and the LC ordering is partially destroyed, probably due to the relatively bigger size of graphene with respect to other carbon nanofillers [45].

### 2.2. Applications of the Carbon-Based LCE Composites 

The incorporation of carbon nanomaterials into the LCE matrix was often motivated by the possibility of extending the actuation mechanisms of LCE-based composite systems in view of possible applications as artificial muscles and electro-active actuators. For instance, the swollen LSCE/carbon-black-integrated layer systems described by Chambers et al. [36,37,38,39] showed actuation properties via resistive “Joule” heating under several cycles of direct current (DC) electric power. When a square-wave current–voltage function is applied to the reprocessed LSCE film, the length of the film decreases, reaching the isotropic (paranematic) phase (actuation state: L_0_) due to the “Joule heating effect”. When the current is off, the film recovers its pristine length assumed at room temperature (relaxed state: L). A maximum elongation of 50% is obtained and the process is reproducible after hundreds of cycles [39]. This value of elongation is similar to that obtained by thermo-mechanical measurements in standard LSCEs, showing that the presence of a carbon-black layer does not prevent the actuation mechanism. 

**Figure 5:**
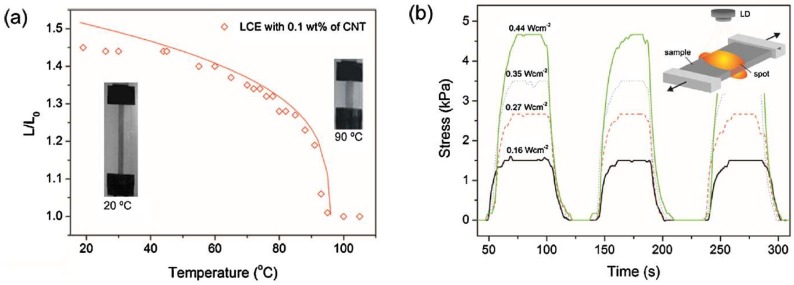
Actuation curves of a stretched LCE sample containing 0.1 wt% of embedded carbon nanotubes (CNTs). (**a**) Contraction ratio (L/L_0_) under uniform heating measured as a function of temperature. Pictures in the inset demonstrate the contraction/elongation phenomenon at the mesophase transition. (**b**) Actuation-stress curves measured under the application of various power densities. The inset presents the scheme of the set-up used for testing. Adapted with permission from the authors of [43]. Copyright © 2018 Elsevier.

A completely new actuation process was observed in the case of LCE-based composites prepared with functionalized CNTs, as reported by Marshall et al. [40]. In this case, the reversible and controllable shape variation of the LCE-composite films is obtained when light is used as actuation stimulus. The photo-actuation of these composites is maximum in the infrared-visible region due to the selective absorption of these wavelengths by the CNTs. In Figure 5a, a typical thermo-mechanical behavior of an LCE-based composite with 0.1 wt% of functionalized CNTs is reported, while Figure 5b illustrates the stress measured on the same system when a uniform light excitation of 620-nm wavelength at various power densities is applied [43].

The use of various concentrations of functionalized CNTs in the LCE matrix is indeed the basis of several applications in the field of micro-optical devices [40,41,42,43], Braille-based displays [56], solar-energy harvesting [57], and tactile and piezoelectric devices [42,58]. The possibility of using LCE composites as light concentrators and heat collectors was recently demonstrated by showing the artificial heliotropism for solar cells, utilizing fiber-network/SWCNT/LCE actuators [57]. These systems can be directly driven by sunlight instead of relying on other power-consuming components for tracking the sun and actuation. Further studies are in progress to optimize several parameters, such as the contraction ratio, the threshold of response to the sunlight, and the loading capability. Another concrete application is related to tactile and haptic displays [56,58], where micro-patterning techniques were used to create arrays of dots able to change their shape either due to LED illumination or variations in temperature.

## 3. LCE-Based Composites with Other Nanomaterials 

In addition to the large use of carbon-based nanostructured materials as fillers and dopants of LCE systems, several researches report LCE-based composites prepared using a wide variety of nanomaterials, such as ferroelectric ceramic nanoparticles [59,60], conductive molybdenum-oxide nanowires [61], magnetic iron-oxide nanoparticles [62,63,64,65], and gold nanomaterials [66,67,68]. In the following sections, the synthesis, properties, and applications of these composites are reviewed. 

### 3.1. Preparation of Nanomaterial-Based LCE Composites

**Figure 6:**
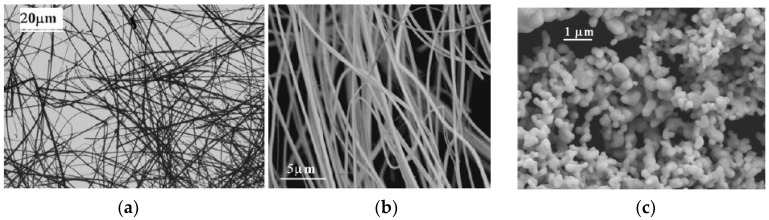
Structured nanomaterials used to prepare LCE-based composites. (**a**) TEM image of the MoO_3−*x*_ nanowires, and (**b**) SEM image of the MoO_3−*x*_ nanowires, revealing their electrical conductivity under 10-keV conditions. Adapted with permission from the authors of [61]. Copyright © 2018 Springer Nature. (**c**) A field-emission SEM picture of PbTiO_3_ quasi-spherical nanoparticles with an average diameter of 100 nm. Adapted with permission from the authors of [60]. Copyright (2018) American Chemical Society.

One of the first attempts to produce composite materials combining the thermo-mechanic and thermo-elastic properties of LCEs and the ferroelectricity of nanoparticles was reported in Reference [59], where the possibility of inducing a macroscopic shape deformation using non-spherical ferroelectric nanoparticles aligned along the main direction of monodomain LCE films was described. Several nanostructured materials were chosen as potential candidates to produce electro-mechanical active composites, such as conductive nanowires made of molybdenum oxides (MoO_3−*x*_) [61] (Figure 6a,b), and ferroelectric ceramic nanoparticles, namely lead titanates (PbTiO_3_) [59,60] (Figure 6c).

The preparation method proposed in References [59,60,61] is similar to that adopted by Marshall et al. [40]. The nanomaterials, either ferroelectric nanoparticles or conductive nanowires, were dispersed in toluene at a known concentration, and the pre-polymerization components (polymer chains, monomers, and cross-linkers) were added in stoichiometric amounts before adding the catalyst. The mixture was introduced to the rotor at 75 °C for about 1 h until a gel-like film was obtained, and then, it was hanged with progressive weights inside an oven at 80 °C for about three days in order to get a monodomain LCE-composite stripe. 

**Figure 7:**
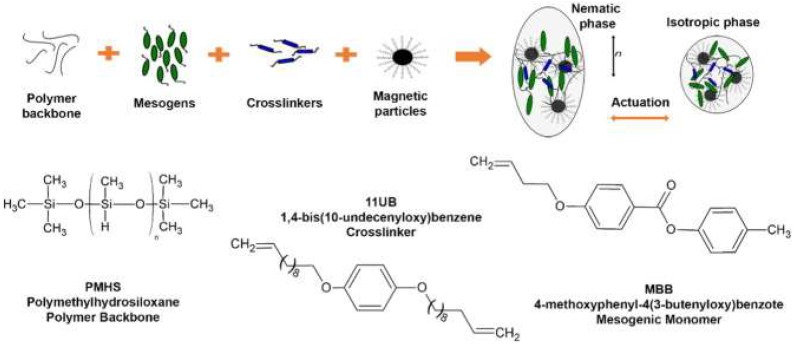
Scheme of the preparation of the composites made from magnetic iron-oxide nanoparticles and a side-chain LCE matrix. Chemical structure of the liquid-crystalline elastomer precursors: the polysiloxane chain (PMHS), the flexible cross-linker (11UB), and the mesogenic monomer (MBB). Reproduced with permission from the authors of [65]. Copyright © 2018 Elsevier.

Similar approaches, which refer to the “two-step” cross-linking reaction proposed by Kupfer et al. [35], were followed to obtain magneto-active LCE composites made of magnetic iron-oxide nanoparticles and side-chain polysiloxane-based LCEs [62,63,64,65]. In most cases, the iron-oxide nanoparticles were made of a core of magnetite (Fe_3_O_4_) surface-coated with oleic acid, N-oleylsarcosine, or analogous functionalization chemicals. A scheme of the preparation and the main chemical components of these magneto-active LCEs, as described in Reference [65], are shown in Figure 7.

**Figure 8:**
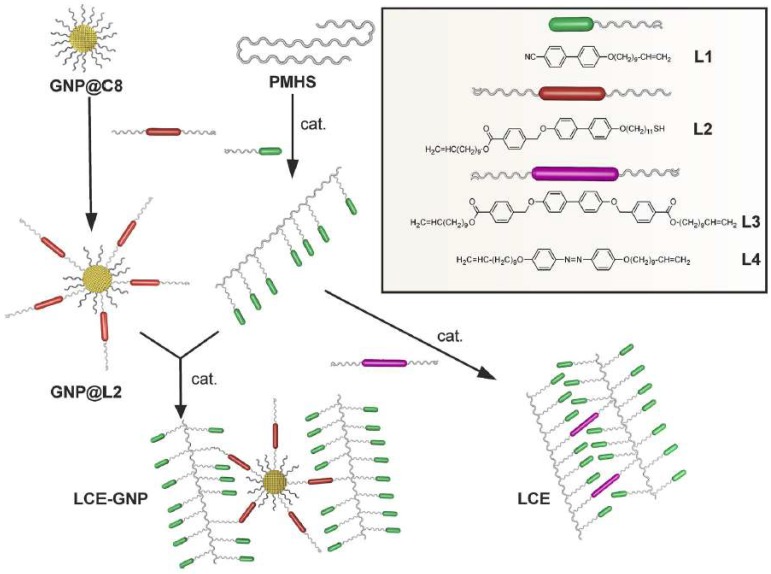
Scheme of the preparation of the LCE-based composites (label LCE-GNP) made of functionalized gold nanoparticles (label GNP@L2) and side-chain LCEs based on polysiloxane chains (PMHS). Mesogenic monomers (label L1) are used as pendant units. Standard LCEs were also prepared with cross-linkers L3 or L4. Reproduced with permission from the authors of [68]. Copyright © 2018 Wiley Online Library.

A new synthetic strategy was recently proposed by Wjjcik et al. [68] to prepare LCE-based composites including gold nanoparticles (GNPs) in the LCE matrix. The approach, shown in Figure 8, consists of the synthesis of gold nanoparticles functionalized with an olefin mesogenic ligand (L2) to obtain metal nanoparticles acting as cross-linking agents (GNP@L2) for the polymer backbones (PMHS). 

Following a synthetic procedure similar to the “two-step” cross-linking reaction, the final LCE-based composites contain metal nanoparticles covalently attached to the polymer chain (LCE-GNP), without any problems of uncontrolled aggregation, ensuring a homogeneous distribution of nanoparticles within the polymer matrix [68].

### 3.2. Alignment of Nanomaterials in the LCE-Based Composites and Orientational-Ordering Properties

As seen for LCE-based composites made of carbon nanomaterials (Section 2), the distribution of nanostructured fillers in the polymer matrix and the degree of orientational order in the LC phase formed by LCE composites are fundamental features in view of the physical properties of these materials. The main experimental techniques used to investigate the alignment of the nanomaterials and the surface morphology of the composites are SEM, TEM, and AFM, while the degree of ordering in the bulk and the orientational order parameters in the mesophase are typically studied by means of X-ray scattering (wide-angle (WAXS) and small-angle (SAXS)) and NMR spectroscopy.

**Figure 9:**
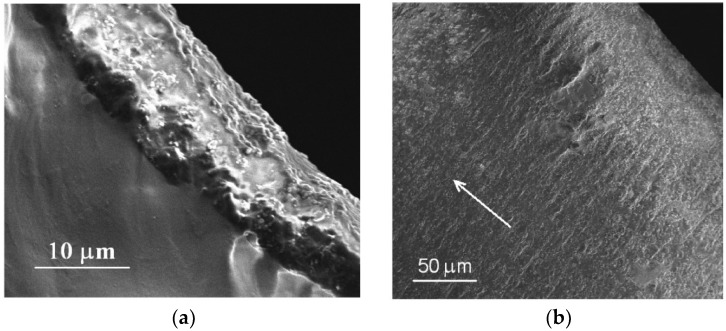
(**a**) Field-emission SEM image obtained on a small piece of a monodomain LCE stripe doped with 5% of PbTiO_3_ nanoparticles. The cross-section of the sample reveals an inhomogeneous distribution of the nanoparticles along the thickness of the stripe. (**b**) Field-emission SEM image of the PbTiO_3_ nanoparticles distributed on the top surface of the composite at 50-μm magnification. The direction of the nematic director (**n**) is indicated by a white arrow. Adapted with permission from the authors of [60]. Copyright (2018) American Chemical Society.

**Figure 10:**
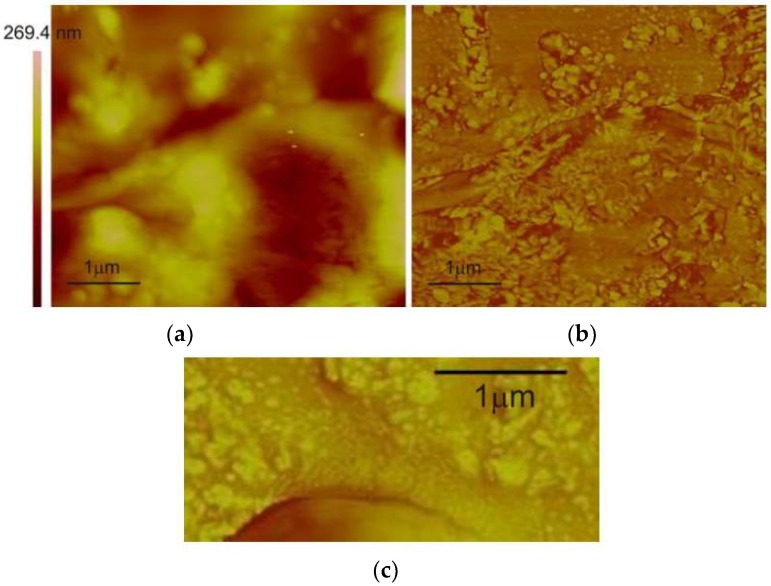
Atomic force microscopy (AFM) topography image (**a**) and phase image (**b**) of the surface of a composite LCE film showing MoO_3−*x*_ nanowires dipping into the polymer matrix. (**c**) Surface of the composite LCE film with MoO_3−*x*_ nanowires showing characteristic undulations. Adapted with permission from the authors of [61]. Copyright © 2018 Springer Nature.

In the case of PbTiO_3_/LCE-based composites [60] and MoO_3−*x*_ nanowire/LCE-based composites [61], the nanomaterials were not homogeneously distributed in the volume of the LCE matrix, but instead, they concentrated on one side of the LCE film. The nanomaterial-rich surface layer was clearly observable by means of SEM and AFM (Figure 9 and Figure 10). In both cases, the nanomaterials were distributed in a layer of about 10-μm depth, showing a particular alignment, visible at the top surface, with respect to the nematic director (parallel to the elongation direction).

As reported in Reference [60], the presence of PbTiO_3_ nanoparticles in various percentages (up to 5 wt%) is responsible for a shift in the transition temperature of a few degrees with respect to the standard LCE prepared with the same procedure and chemical composition. This means that the nanomaterials do not significantly influence the mesomorphic properties. The LCE-based films are indeed transparent, thus indicating a good macroscopic alignment of the nematic directors. However, as shown in Figure 9, the distribution of PbTiO_3_ nanoparticles on the top surface is regular and anisotropic. In fact, the nanoparticles are distributed along parallel stripes perpendicular to the nematic director (**n**) with a typical period of few micrometers.

A similar anisotropic distribution of nanomaterials was observed in MoO_3−*x*_ nanowire/LCE-based composites [61]. Most of the MoO_3−*x*_ nanowires are indeed distributed on the top surface of the composite films. This is due to the fact that, during the first cross-linking step of the “two-step” cross-linking reaction, the nanowires tend to separate from the other pre-polymerization components, as noted in the case of ferroelectric nanoparticles [60], because of the different relative weight. The analysis of SEM and TEM images [61] reveals a uniform alignment of the nanowires along the director **n**, thus indicating that the adopted procedure allowed us to induce a preferred alignment of the nanowires into the top surface of the LCE matrix. Topography and phase AFM imaging modes were used to investigate the surface of composite films. This study indicates that the nanowires are located close to the surface of the LCEs (Figure 10a,b), well-embedded into the surface layer of the polymer matrix. Moreover, characteristic surface deformations are observed on the top surface of the LCE composites (Figure 10c), probably due to Euler strut instabilities typical of adjacent systems having a different Young’s modulus [61]. 

**Figure 11:**
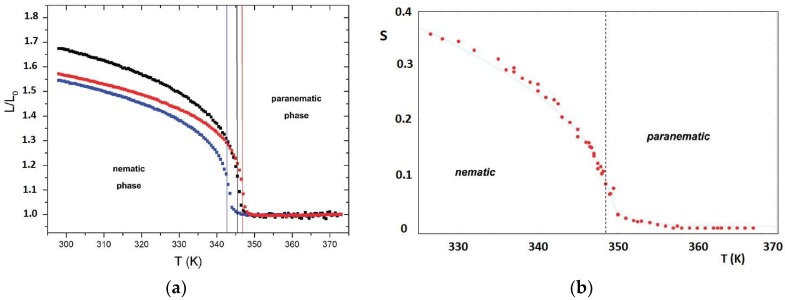
(**a**) Thermo-mechanical behavior of the standard monodomain LCE film (blue symbols), and LCE films doped with 1% (red symbols) and 5% (black symbols) PbTiO_3_ nanoparticles. Temperature transitions between the paranematic and nematic phases are indicated by vertical lines. (**b**) Temperature dependence of the orientational-order parameter (*S*) determined by analyzing the ^2^H NMR quadrupolar splittings in terms of the Landau–de Gennes modified theory, as described in References [21,69,70]. A dashed line corresponds to the transition temperature obtained from the fitting of the orientational order. Adapted with permission from the authors of [60]. Copyright (2018) American Chemical Society.

Despite the inhomogeneous distribution of nanomaterials in the LCE matrix, and the formation of a surface layer with a high density of nanofillers (either MoO_3−*x*_ nanowires or PbTiO_3_ nanoparticles), the thermo-mechanical properties of the LCE-based composite films are similar to those of the pristine LCE materials (Figure 11a). The addition of 1 wt% and 5 wt% of lead-titanate nanoparticles reduces the maximum elongation from 68% to about 55% in both cases [60]. Similarly, the presence of molybdenum-oxide nanowires reduces the maximum elongation of the LCE films by about 10% with respect to standard LCEs [61]. The increased stiffness of the LCE composites, however, has little effect on the mesomorphic properties, except for a slight shift in the paranematic-to-nematic phase transition (see Figure 11a). 

An important aspect of these LCE-based systems is related to the degree of orientational order as the temperature is varied from T > T_N-PN_ to room temperature and vice versa. Deuteron (^2^H) NMR studies of selectively labeled LCE and LCE-based composites were of help in deeply investigating this aspect [21,60,69,70,71,72,73,74]. The temperature dependence of the measured ^2^H NMR spectra, and the trends of the quadrupolar splitting can be analyzed to obtain the local orientational-order parameter, *S(T)*, which indicates the average degree of order of the principal mesophase director (**n**). A typical trend of *S(T)* in an LCE system (standard or composite) is reported in Figure 11b, showing the continuous increase in local order at the paranematic–nematic transition upon decreasing the temperature. The continuous characteristics of the temperature dependence of *S* is a clear indication of the criticality (or super-criticality) of the paranematic–nematic transition [69,70], which can be modeled through a modified Landau–de Gennes theory of the free-energy density of LCE systems. This approach [69,70] gives rise to a complete description of the microscopic properties of LCEs, and it associates the critical characteristics of the mesophase transition to the presence of internal mechanical fields in the LCE network, quantified by a parameter, g. According to this analysis, the presence of nanomaterials in the LCE matrix increases the parameter g to values five times larger than the critical value (g_c_) shifting the mesophase transition from a critical to a supercritical one.

**Figure 12:**
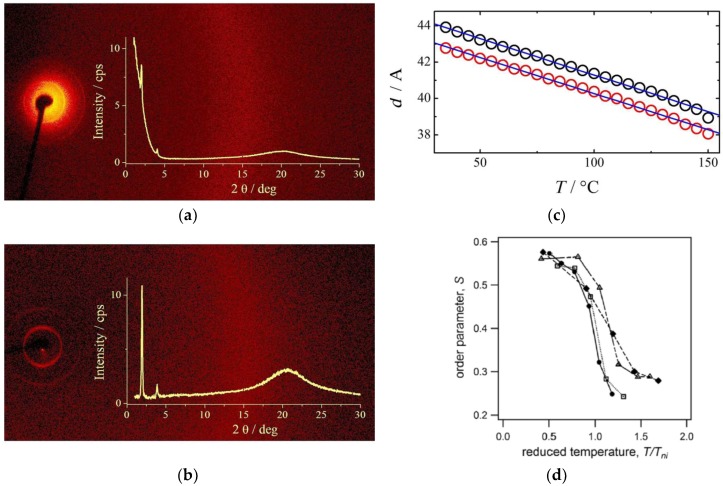
(**a**,**b**) Two-dimensional (2D) X-ray diffraction (XRD) patterns taken in the broad-angle range for smectic phases of polymers. (**a**) LCE-GNP with a hybrid gold-nanoparticle cross-linker; (**b**) standard LCE prepared with L3 as the cross-linker. (**c**) Temperature dependence of smectic-layer thickness for LCE-GNP (black circles) and LCE (red circles). Reproduced with permission from the authors of [68]. Copyright © 2018 Wiley Online Library. (**d**) Order parameter (*S*) as a function of the reduced temperature (*T/T_ni_*) of LCE composites containing various percentages of iron-oxide nanoparticles, as obtained using small-angle X-ray scattering (SAXS) measurements: LCE pristine matrix (●), 0.2 wt% (□), 0.5 wt% (Δ), and 0.7 wt% (◆) nanoparticle content. Reproduced with permission from the authors of [65]. Copyright © 2018 Elsevier.

Moreover, as shown in Figure 11b, the value of the orientational order (*S*) at room temperature is comparable with that measured in standard LCEs [60,69,70,71,72]. Relatively high values of the orientational-order parameter (*S*), obtained from X-ray diffraction analysis, were also found in magneto-active LCE-based composites prepared with iron-oxide nanoparticles [65]. Another common technique used to determine the orientational-order parameter in LCEs is indeed based on X-ray diffraction (XRD) as reported in Figure 12. LCEs with and without the inclusion of magnetic nanoparticles, for instance, have *S* values between 0.5 and 0.6 at low temperatures (Figure 12d). The high value of *S* in the ordered phase indicates that the degree of order in the low-temperature phase is not significantly reduced by the addition of the nanoparticles [65]. The X-ray patterns can give information not only about the average order, but also about the homogeneity of distributions, and about the nature of the mesophases. Both Figure 12a,b evidence the occurrence of lamellar (smectic) structures for two gold-nanoparticle LCE composites, namely LCE-GNP1 and LCE-GNP2 [68], with layer spacings in the range of 39–44 Å (Figure 12c), thus indicating the presence of a partially interdigitated structure. The diffuse XRD signal observed at high angles, corresponding to 4.5 Å, reflects the liquid-like order of mesogens within the smectic layers. Moreover, temperature-dependent X-ray measurements (upon cooling and heating the samples) show that the gold nanoparticles remain well-dispersed in the polymer network, with a highly homogeneous distribution without the formation of nanoparticle agglomerations.

The polymer conformation and chain anisotropy of liquid-crystalline elastomers were also the object of several studies, based on a combination of X-ray techniques (namely SAXS and WAXS) [75,76] and small-angle neutron scattering (SANS) [77,78,79]. However, to our knowledge, no such studies were performed to investigate the ordering and structural properties of nanomaterial/LCE composites. 

### 3.3. Actuation Properties and Applications of Nanomaterial-Based LCE Composites

**Figure 13:**
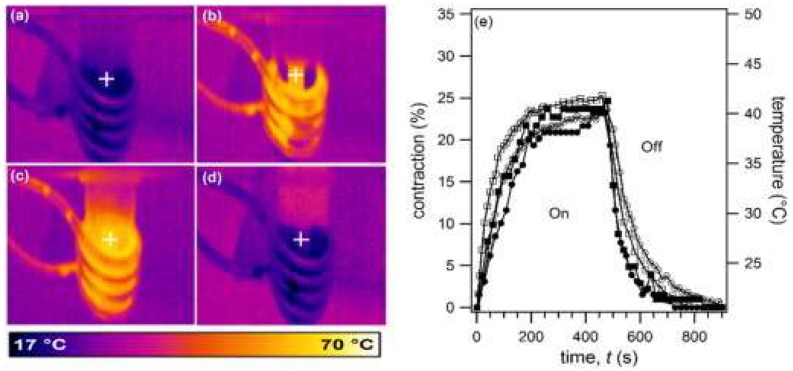

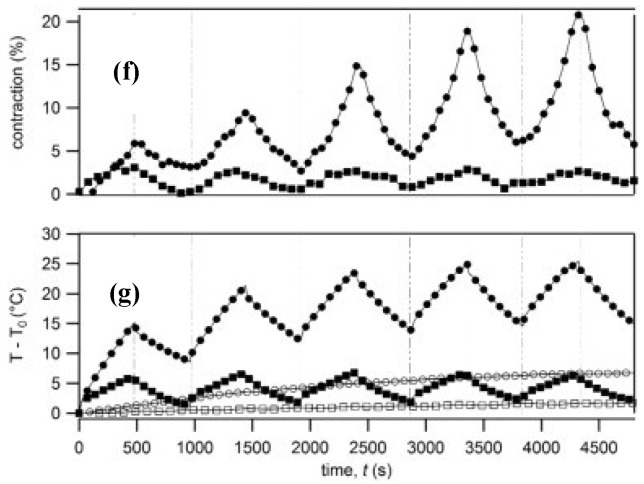
Thermal images of LCE-based composites with 0.5 wt% of iron-oxide nanoparticles when subjected to an alternating magnetic field of 48.24 kA/m and 298-kHz oscillating frequency at (**a**) 0, (**b**) 27, (**c**) 159, and (**d**) 926 s. (**e**) Superposition of the temperature (unfilled symbols) and contraction (filled symbols) profiles of the LCE composites with 0.5 wt% (●) and 0.7 wt% (■) of iron-oxide nanoparticles obtained in a magnetic-actuation experiment. Crosses in thermal images represent the reference points where the temperature data were collected using the infrared camera’s software. Mechanical deformation (**f**) and temperature increments (**g**) of LCE-based composites with 0.5 wt% of iron-oxide nanoparticles in response to an applied alternating magnetic field of 34.73 kA/m at 298 kHz over five eight-minute on/off cycles submerged in water (■) and in a model cell-growth medium (labeled DMEM; ●). Filled and unfilled symbols in the bottom figure correspond to temperatures measured close to the surface of the elastomer and at the bottom of the reservoir, respectively. T_0_ corresponds to the initial temperature at *t* = 0. Reproduced with permission from the authors of [65]. Copyright © 2018 Elsevier.

As seen in the previous section, the addition of nanomaterials does not alter the thermo-mechanical behavior, preserving the ability of the LCE-based composites to act as shape-memory actuators subject to temperature variations. However, in some cases, the nanostructured dopants induce new properties, such as the magneto-mechanical response of LCE-composite films prepared with iron-oxide nanoparticles [62,63,64,65]. Kaiser et al. [62] reported a reversible magnetic-field-induced shape change in LCE-doped systems under on/off cycles of various electromagnetic-field amplitudes (from 8.5 to 42.6 kA/m), reaching a contraction of the starting composite films comparable to the thermally induced contraction. Herrera-Posada et al. [65] confirmed the potential of LCEs doped with 0.5 and 0.7 wt% oleic-acid-coated iron-oxide nanoparticles as magneto-active elastomers. In Figure 13a–d, the temperature evolution of the magnetic elastomers determined from thermal infrared images is reported, while the superposition of the instant temperature of the magneto-active elastomers during actuation, and the contraction percentage profiles are shown in Figure 13e. The application of an alternating field of 48.24 kA/m and an oscillating frequency of 298 kHz causes relaxation and hysteresis phenomena in the magnetic nanoparticles, leading to energy dissipation or heat loss [65]. This mechanism is at the origin of the thermally driven isotropic–nematic transition from the heat generated by the magnetic nanoparticles. 

In this work [65], the possibility of using magnetic iron-oxide nanoparticle/LCE composites in biological environments was investigated by studying the magneto-mechanical response in deionized water and in a model cell-growth medium (labeled DMEM). The time profiles of the contraction of LCE-based composites, and the fluid temperature close to the films and at the bottom of the container are reported in Figure 13f,g, respectively. As a main result, the LCE-composite systems have a lower contraction in deionized water than in air, because the surrounding water acts as a heat sink which dissipates the heat generated by the magnetic nanoparticles in the LCE matrix. When DMEM fluid is used instead of water, the magneto-mechanical response increases during each successive cycle, and this is probably due to the presence of salts and electrolytes. Even though the magnetically-induced contractions in DMEM are lower than in air, they are reproducible, and the temperature of the fluid remains lower than 41 °C.

Additional results reported in Reference [65] demonstrate that collagen treatment applied to the surface of LCE films, with and without magnetic nanoparticles, allowed for the effective attachment and proliferation of a special kind of fibroblast. This study opens up several new applications in the field of bio-medical devices, overcoming the limitations due to the inherent hydrophobicity of polysiloxane matrices.

**Figure 14:**
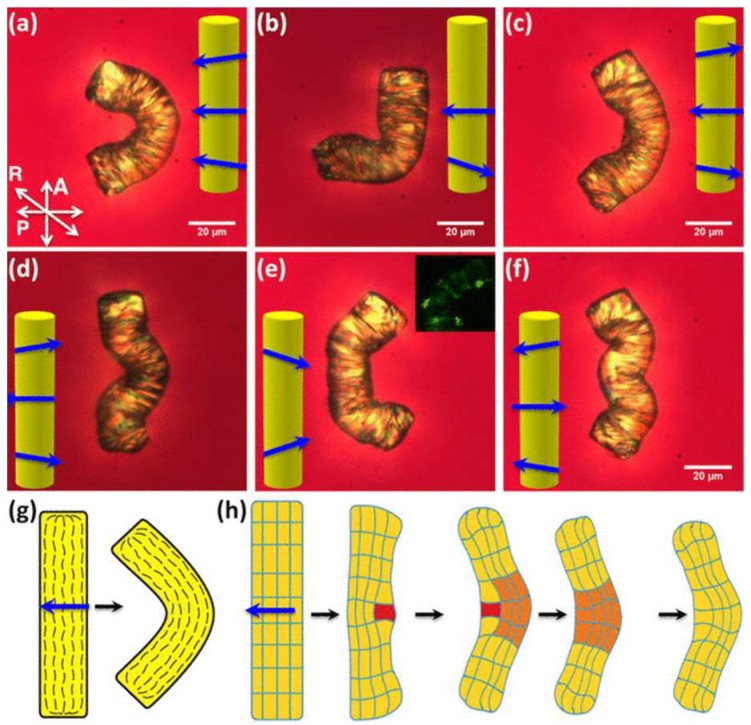
(**a**–**f**) Examples of reversible morphing of LCE micro-particles including gold nanoparticles, shaped by means of unidirectional laser-beam scanning along the blue arrows. The inset in (**e**) shows a typical coherent anti-Stokes Raman scattering polarizing microscopy image of an LCE gold-nanoparticle-doped micro-particle that was bent using a scanning laser beam. (**g**) Scheme of director distributions in the LCE micro-particle during the bending induced by the laser beam. (**h**) Scheme showing the effect of local manipulation of LCE orientational ordering by a scanned laser beam along the direction marked by the blue arrow. The scanning causes nonreciprocal unidirectional motion of a molten region (red), and a modification of the LCE polymeric network in the vicinity of the “hot” scanning region (orange), eventually leading to the stable modification of the particle shape, persisting after the laser is turned off. Reproduced with permission from the authors of [66]. Copyright © 2018 AIP Publishing.

The addition of gold nanoparticles to LCEs was investigated by several groups [66,67,68,80], with the aim of producing photo-thermal effects related to the localized surface plasmon resonance of gold nanoparticles. Several works [66,67,80] proposed the doping of LCE micro-particles or LCE micro-pillars with gold nanoparticles to induce shape deformations, rotations, and/or translations using optical manipulation. Liu et al. [80] fabricated micro-sized polyacrylate-based LCEs doped with 1 wt% of gold nanoparticles (and gold nanorods), and they were able to induce a maximum strain of 30% of the initial LCE length using a laser of 635-nm wavelength. Sun et al. [66] used a different synthetic approach based on a soft lithography molding technique to produce micrometer-sized cylindrical LCE actuators. They discussed the mechanism of manipulation of the shape of these colloidal birefringent elastomeric micro-particles [66] using infrared laser beams and polarized-light optical tweezers. In this work [66], a 1064-nm laser source was selected, since this wavelength is about double that of the maximum absorption wavelength of the pristine gold nanoparticles, thus allowing an efficient two-photon absorption process. Moreover, infrared light has several advantages with respect to visible light, since it can penetrate into the bulk of LCEs, reducing the scattering. A special profile of the local nematic director (**n**) was achieved by means of nonlinear optical polarizing microscopy, including coherent anti-Stokes Raman scattering polarizing microscopy (Figure 14). The shrinking of the LCE micro-particle’s length and an increase in its diameter are normally observed when heated, due to the increase in local temperature across the nematic–isotropic phase transition. When the LCE micro-particles are doped with gold nanoparticles, the same effect can be observed due to photo-thermal transfer of energy into heat. Interestingly, the shape variation of the micro-cylinder quickly relaxes back to the original shape within about one second. As demonstrated by Sun et al. [66], optical manipulation of LCE micro-particles doped with gold nanoparticles can be achieved with robust and reproducible spatial (translational and rotational) control, opening up an opportunity for new technological applications.

A very recent work [81] deals with anisotropy shape deformation of swollen LCE micro-pillars doped with gold nanoparticles, with the possibility of using either UV or IR light stimuli. Additional potential applications of gold-nanoparticle-doped LCEs range from contact-free actuation of colloidal micro-particles to micro-fluidics and opto-mechanics, as also supported by recent theoretical investigations [82]. Very recent works on nanostructured LCE-based materials were published aiming to introduce copper-sulfide nanoparticles [83] and silver nanoparticles [84] into the LCE matrix to induce photo-actuation mechanisms. 

## 4. LCE-Based Bilayer Composites 

Another field of research related to LCE composites concerns the preparation of bilayered or multilayered systems obtained by coupling monodomain LCE films, mainly prepared according to the “two-step” cross-linking procedure [35], and one or two layers of conductive materials deposited onto the top surface of LCEs. These bilayered (or multilayered) systems were the object of several experimental and theoretical works in the past six years [85,86,87,88,89,90,91,92,93,94,95]. Several new technological applications were explored in detail based on unique phenomena characterizing the LCE-based bilayer composites, such as bending actuation [85,87,89,91,92,93,94,95] and surface wrinkling [85,86,88,90,92,93,94], as described in the sections below. 

### 4.1. Preparation of Bilayered (or Multilayered) Systems 

**Figure 15:**
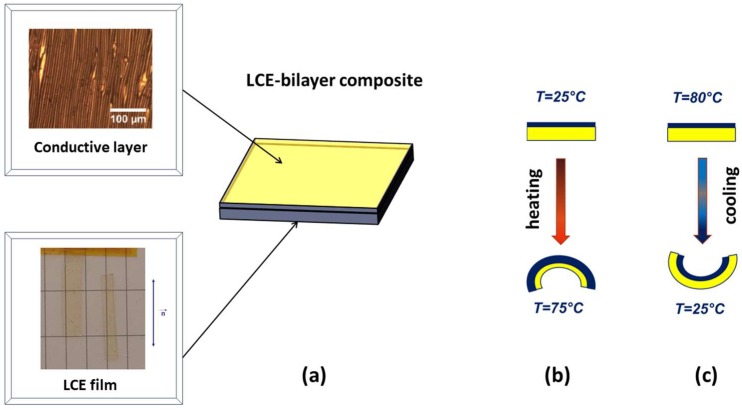
(**a**) Scheme of the LCE-based bilayer composites made using monodomain side-chain LCE films and a nanostructured conductive layer [92,93]. Different temperature-induced bending geometries of the LCE-based bilayers depending on the temperature of deposition of the conductive layer: (**b**) T << T_N-PN_ (T = 25 °C), LCE in the nematic (ordered) phase; and (**c**) T > T_N-PN_ (T = 80 °C), LCE in the paranematic or isotropic (disordered) phase.

The first bilayer LCE-systems were produced by depositing conductive nanostructured thin polymer layers on the top surface of standard polysiloxane-based LCE films (see the general schemes reported in Figure 15). Agrawal et al. [90] used nanolayers of poly(styrene) (PS), while Domenici, Greco et al. [86,87,88] chose the poly(3,4-ethylenedioxythiophene):poly(styrenesulfonate) (PEDOT:PSS) conductive polymer. 

Nanoscale PS thin films were prepared [90] by spin-casting a PS solution on a silicone-based plate, cleaned by UV/ozone treatment, placed on the top surface of LCE films, before being put in water at a known temperature, pealed off, and dried in vacuum. PS layers of varying thickness were prepared, ranging from 30 nm to 400 nm, while the LCE-film thickness was typically on the scale of 0.3/0.4 mm [90]. 

In the case of PEDOT:PSS/LCE bilayers, several strategies were optimized [85,86,87,88,89]. The PEDOT:PSS material was chosen for its excellent properties of conductivity, and mechanical and chemical stability, and for the possibility of fabricating nanolayers with very high control and reproducibility [96,97]. The monodomain polysiloxane-based LCE films were exposed to air plasma to improve the wettability of their surface by aqueous solutions. A filtered dispersion of PEDOT:PSS in water was deposited with a micropipette over the LCE films. Homogeneous layers of PEDOT:PSS of varying thickness were prepared using a spin-coating deposition, and were then dried at room temperature [88]. An improved approach [97], used to enhance the conductivity of the PEDOT:PSS nanolayers, was adopted via the introduction of dimethylsulfoxide (DMSO) in the preparation procedure. To fabricate electro-active LCE-based bilayers, the LCE films were exposed to plasma, as described above, and thin copper wires were placed on the top surface to provide a good electrical contact for applying voltage to the actuator. Deposition of the DMSO-doped PEDOT:PSS nanolayer was done by drop-casting of the solution onto the LCE stripes and the wires, resulting in a good adhesion and electrical contact between the wire and the conductive layer. In all cases [85,86,87,88,89], the PEDOT:PSS material was deposited onto the surface of the LCE films at room temperature, i.e., in the nematic phase (Figure 15b). 

Zupancic et al. [92] reported the preparation of bilayers (and multilayers) of LCE films reprocessed with conductive gold nanolayers. The deposition of gold nanolayers using sputtering technology was performed after the creation of an adhesion layer of chromium on the LCE surface. The average thickness of the gold layer, as determined from SEM investigations, was on the scale of ~100 nm, while the Cr adhesion layer was about a few nanometers thick. Single-sided samples, with a layer deposited on one side of the LCE film, and double-sided samples, with layers deposited on both sides of an LCE film, were prepared using this procedure [92]. 

As reported in several works, whether experimental and theoretical [88,89,90,91,92,93,94], the final physical properties of the LCE-composite bilayers depend on two parameters: (1) the ratio between the nanolayer thickness (d_nl_) and the LCE thickness (d_LCE_), and (2) the temperature of the conductive nanolayer deposition. The ratio (d_nl_)/(d_LCE_) is a crucial parameter, since, as discussed in the next section, two different physical behaviors can be observed: bending actuation or surface wrinkling. The temperature of deposition is also an important parameter. If the layer is deposited on the LCE film at temperatures below the isotropic–nematic transition, namely in the elongated (ordered) state, or above the isotropic–nematic transition, namely in the contracted (disordered) state, different geometries are observed. In Figure 15b,c, for instance, a different bending geometry of the bilayer systems is obtained depending on the temperature of the nanolayer deposition.

### 4.2. Bending Actuation and Surface Micro-Wrinkling Phenomena 

**Figure 16:**
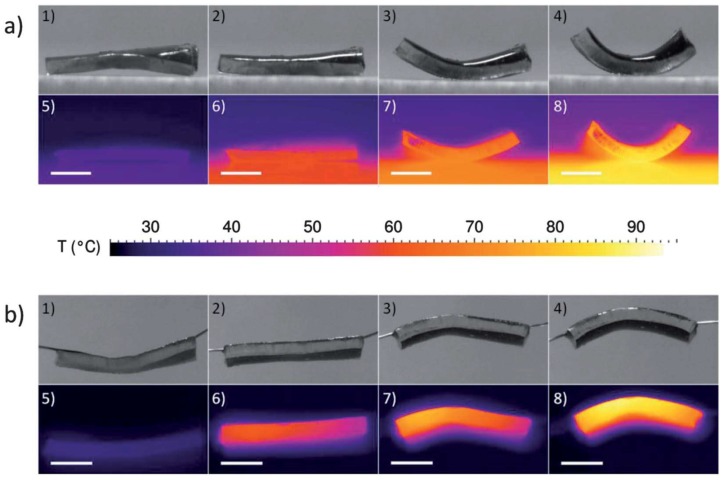
Bending actuation of LCE/ poly(3,4-ethylenedioxythiophene):poly(styrenesulfonate) (PEDOT:PSS) bilayer actuators. (**a**) Direct heating experiment using a Peltier element. Camera frames taken (**1**) at room temperature, (**2**) at the nematic–isotropic transition onset, (**3**) during the transition, and (**4**) at a temperature higher than the temperature transition. (**5**–**8**) Corresponding thermal infrared camera images. (**b**) Joule heating experiment. Camera frames taken at (**1**) room temperature, (**2**) at the nematic–isotropic transition onset, (**3**) during the transition, and (**4**) at a temperature higher than the temperature transition. (**5**–**8**) Corresponding thermal infrared camera frames (scale bar: 1 mm). Reproduced with permission from the authors of [89]. Copyright (2018) The Royal Society of Chemistry.

The bending actuation of bilayer systems was the object of several investigations [98,99,100,101,102,103]; however, the fabrication of robust actuators, with good electrical contacts, and reproducible and durable systems after thousands of on/off cycles still represents an unresolved issue. One of the main problems is related to the compatibility (chemical and physical) between the two layers of different materials and their intrinsically different stiffness. As demonstrated by Greco et al. [88,89], excellent results were obtained in the case of LCE/PEDOT:PSS bilayers. PEDOT:PSS indeed has a Young’s modulus and Poisson’s ratio very similar to those of the LCE films. This aspect is a great advantage with respect to other bilayered systems [91,92], since PEDOT:PSS acts as an incompressible “skin” during the temperature-induced actuation, inducing the elongation/contraction of the LCE films. The perfect adhesion of the PEDOT:PSS nanolayer to the surface of the LCE results in the bending of the whole bilayered structure when the temperature is varied across the nematic-to-isotropic phase transition. In Figure 16a, the thermal-bending actuation of an LCE/PEDOT:PSS bilayer is reported. This particular sample was prepared by depositing the PEDOT:PSS layer at T = 25 °C (T << T_N-PN_, with T_N-PN_ = 73.7 °C), and a ratio (d_nl_)/(d_LCE_) of about 20 μm/300 μm. The bilayered system was placed on top of a Peltier cell with the PEDOT:PSS-coated side facing down. The variation in temperature induces the bending actuation, and simultaneously, the temperature distribution across the sample is monitored using a thermal infrared-imaging camera. 

A similar experimental set-up was realized to study the “Joule” heating-based actuation of an LCE/PEDOT:PSS bilayer sample, prepared including thin copper wires embedded in the PEDOT:PSS layer, as described in the previous section. The final PEDOT:PSS thickness was about 15 μm, and the bilayer resistance at room temperature was about 60 Ω. The system was connected with an electrical power supply, and several cycles of voltage were applied (V = 0–1.3 V). The bending actuation of the bilayer was studied by recording a video with visible and thermal imaging cameras. Selected frames are reported in Figure 14b. The very good thermal conductivity of the PEDOT:PSS nanolayers ensures a uniform heating to the LCE. In both experiments (thermal-bending actuation and “Joule” heating-induced actuation), the temperature of the LCE layer is almost uniform. The two mechanisms of actuation, thermally induced and electrically induced, are indeed very correlated, since in both cases, the driving force is given by the occurrence of a transition between a disordered state (not aligned LCs) to an homogeneously ordered state (LC moieties aligned along the elongation direction). Such a mesophase transition is an intrinsic property of the LCE thin films. 

The bending actuation of LCE/PEDOT:PSS bilayer samples was studied using analysis of the curvature-versus-temperature trend, and a simple model was developed [89] taking into account both geometrical and elastic properties of the two components of the LCE-based composites. The very good electrical control of the bending actuation of LCE/PEDOT:PSS bilayers opens up many practical applications in the field of soft robotics, micro-robotics, medical devices, and space robotics. Moreover, LCE-based composites are promising biomimetic materials, since their typical stress and strain values are similar to those of natural skeletal muscle, and their mechanical performance is comparable to that of dielectric elastomer actuators [6,88,89,93]. 

As reported in several studies of LCE-based composite bilayers [86,87,88,89,90,91,92], if the ratio (d_nl_)/(d_LCE_) is lower than a critical value (which depends on the chemistry of the conductive nanolayers), bending actuation is not observed. Instead, micro- or nanostructured surface wrinkling is obtained with the possibility of fine-tuning their geometrical properties. Surface instabilities are the object of recent investigations to better understand their formation in natural systems (such as wrinkles originating in the aging human skin and in dried fruits), and to utilize the self-organization surface patterning in a controllable way to measure thin-film properties [92]. The formation of wrinkles when a two-layered system, made of materials with different elastic properties, is stretched or compressed is a known phenomenon [93,102,103,104]. In the case of LCE-based composite layers, however, the possibility of generating periodic, tunable, and reversible surface patterns opens up new intriguing applications. 

**Figure 17:**
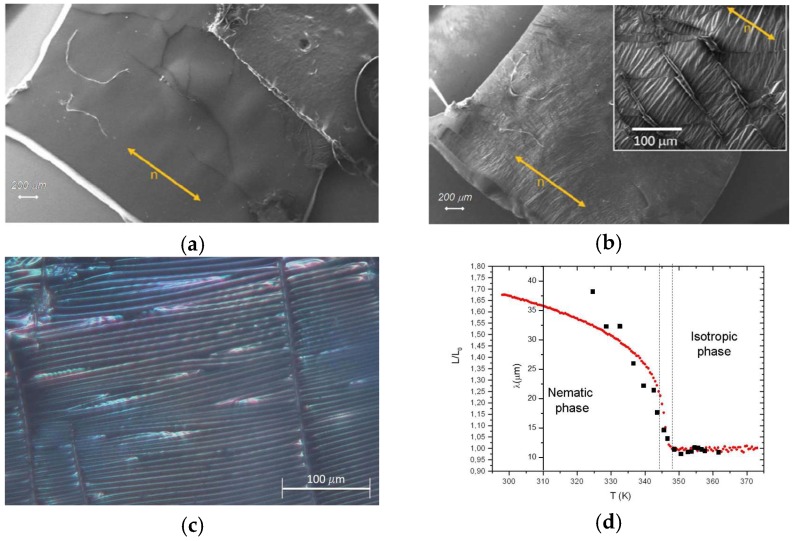
SEM images showing the surface of the LCE/PEDOT:PSS composite (**a**) before and (**b**) after heating at T ≈ 73 °C. Direction defining nematic director (**n**) alignment is shown as an orange arrow. Inset at left of image (**b**) shows a closer view of the surface in which uniaxial microwrinkles are formed and organized in a domain-like arrangement. (**c**) Image of the surface of the LCE/PEDOT:PSS composite, observed using an optical microscope. The temperature of the sample is about 75 °C, i.e., in the isotropic phase. Horizontal and parallel wrinkles, as well as vertical breaks, are clearly visible. (**d**) Wrinkle wavelength (λ) as measured by observing several images of PEDOT:PSS/LCE composites using an optical microscope (black squares) during the second cooling process, in the temperature range of 90–52 °C. Thermo-mechanic elongation (L/L_0_) of the pristine LCE film (red circles) during the second cooling run. Vertical dot lines are added in order to put in evidence the isotropic–nematic phase transition. Reproduced with permission from the authors of [88]. Copyright (2018) Taylor & Francis.

Greco et al. [88] showed that if the PEDOT:PSS nanolayer thickness is below 200 nm, micro-wrinkle formation is observed when the temperature is changed across the isotropic–nematic transition. On the contrary, PEDOT:PSS layer thickness around 5–20 μm produces bending actuation. The formation of uniaxially aligned micro-wrinkles can be observed in the LCE/PEDOT:PSS bilayer sample after several thermal cycles, showing reproducible variations in wrinkle periodicity. In Figure 17a,b, SEM images show the surface morphology before and after heating the sample in the isotropic (contracted) phase. Parallel micro-wrinkles are visible in Figure 17b, showing a particular organization with respect to local domain arrangement. The direction of parallel wrinkles is perpendicular to the nematic director (**n**), corresponding to the direction of compression/elongation at the isotropic–nematic transition temperature. As also seen using optical microscopy (Figure 17c), vertical breaks appear when the sample is heated for the first time in the isotropic phase. These vertical ruptures define the domain boundaries, and they do not change during several cycles of heating/cooling, nor do they affect the micro-wrinkling phenomenon. In Figure 17d, the temperature trend of the micro-wrinkle wavelengths is reported, and it overlaps with the thermo-mechanical elongation/contraction (L/L_0_) behavior of the LCE matrix. Interestingly, the larger variation in the micro-wrinkle wavelengths (from 10 to 40 microns) occurs in a relatively small temperature range, and it is fully reproducible after several cycles [88]. Very similar results were reported by Agrawal et al. [90] in PS/LCE composite bilayers, where the formation of both vertical and horizontal wrinkle patterns is obtained by changing the temperature of PS nanolayer deposition. The reproducible micro-wrinkling behavior is particularly promising for optical applications, such as the formation of tunable optical gratings and new devices for measuring mechanical properties in thin films [104,105,106]. 

## 5. General Conclusions and Perspectives

In this review, the development of a recent expanding field related to liquid-crystalline elastomers combined with nanostructured materials to form composite systems was overviewed, focusing on the main synthetic strategies, their unique physico-chemical properties, and their potential technological applications. Most intriguing properties of these LCE-based composites derive from their peculiar self-assembling behavior (i.e., the orientational order, and the nanomaterial alignment and distribution within the LCE matrix), as well as from the physical properties of the chosen nanomaterials (i.e., electric conductivity, photo-absorption, and ferroelectric and magnetic properties). So far, reversible and controllable shape deformation induced by thermal stimuli represents the driving mechanism at the basis of future applications of LCE-based composites. However, new promising mechanisms based on photo-actuation, magneto-actuation, and “Joule”-heating electrically driven actuation were observed and are described in this review, taking into account very recent works [107,108,109,110,111,112]. Future developments of these polymer composite materials will probably expand to the field of biological applications and biomimetics [65,113,114,115,116], optical tunable gratings [117,118,119], three-dimensional (3D) printing and 3D morphing technologies [20,32,120,121,122], and flexible stretchable electrodes [123,124].

## Abbreviations

AFM	Atomic force microscopy
C60	Fullerene
CNT	Carbon nanotube
GNP	Gold nanoparticle
DLS	Dynamic light scattering
LC	Liquid crystal
LCE	Liquid-crystal elastomer
LCE@GNP	Liquid-crystal elastomer composite including gold nanoparticles
LCP	Liquid-crystal polymer
LMMLC	Low-molecular-mass liquid crystal
LSCE	Liquid single-crystal elastomer
MWCNT	Multi-walled carbon nanotube
MEMS	Micro-electromechanical systems
N	Nematic phase
NEMS	Nano-electromechanical systems
NMR	Nuclear magnetic resonance
PEDOT	Poly(3,4-ethylenedioxythiophene)
PEDOT:PSS	Poly(3,4-ethylenedioxythiophene):poly(styrenesulfonate)
PCNT	Polymer composite made of CNT
PC60	Polymer composite made of C60
PG	Polymer composite made of graphene
PN	Paranematic phase
PS	Poly(styrene)
S	Orientational-order parameter
SAXS	Small-angle X-ray scattering
SANS	Small-angle neutron scattering
SEM	Scanning electron microscopy
SmA	Smectic A phase
SWCNT TEM	Single-walled carbon nanotube Trasmission electron microscopy
T_N-PN_	Temperature of the transition from the nematic to the paranematic phases
WAXS	Wide-angle X-ray scattering
XRD	X-ray diffraction
